# Bee venom phospholipase A2 ameliorates amyloidogenesis and neuroinflammation through inhibition of signal transducer and activator of transcription-3 pathway in Tg2576 mice

**DOI:** 10.1186/s40035-019-0167-7

**Published:** 2019-10-02

**Authors:** Hyeon Joo Ham, Sang-Bae Han, Jaesuk Yun, In Jun Yeo, Young Wan Ham, Se Hyun Kim, Pil-Hoon Park, Dong-Young Choi, Jin Tae Hong

**Affiliations:** 1 College of Pharmacy and Medical Research Center, Chungbuk National University, Osongsaengmyeong 1-ro, Osong-eup, Heungdeok-gu, Cheongju, Chungbuk 28160 Republic of Korea; 2 Department of Chemistry, Utah Valley University, 800 W University Pkwy, Orem, UT 84058 USA; 3 INISTst Co., LTD, 767, Sinsu-ro, Suji-gu, Yongin-si, 16827 Gyeonggi-do Republic of Korea; 4 College of Pharmacy, Yeungnam University, 280 Daehak Road, Gyeonsan, Gyeongbuk, 38541 Republic of Korea

**Keywords:** Bee venom phospholipase A2, Alzheimer’s disease, Amyloidogenesis, Neuroinflammation, STAT3

## Abstract

**Background:**

Neuroinflammation and accumulation of β-amyloid (Aβ) play a significant role in the onset and progression of Alzheimer’s disease (AD). Our previous study demonstrated that signal transducer and activator of transcription-3 (STAT3) plays a major role in neuroinflammation and amyloidogenesis.

**Methods:**

In the present study, we investigated the inhibitory effect of bee venom phospholipase A2 (bvPLA2) on memory deficiency in Tg2576 mice, which demonstrate genetic characteristics of AD and the mechanism of its action at the cellular and animal level. For in vivo study, we examined the effect of bvPLA2 on improving memory by conducting several behavioral tests with the administration of bvPLA2 (1 mg/kg) to Tg2576 mice. For in vitro study, we examined the effect of bvPLA2 on amyloidogenesis and neuroinflammation by treating bvPLA2 on LPS-activated BV2 cells.

**Results:**

We found that bvPLA2 alleviated memory impairment in Tg2576 mice, as demonstrated in the behavioral tests assessing memory. In the bvPLA2-treated group, Aβ, amyloid precursor protein (APP), and β-secretase 1 (BACE1) levels and β-secretase activity were significantly decreased. Expression of pro-inflammatory cytokines and inflammation-related proteins decreased in the brain of bvPLA2-treated group, whereas anti-inflammatory cytokines increased. In addition, bvPLA2 reduced STAT3 phosphorylation in the brains of the bvPLA2-treated group. At the cellular level, bvPLA2 inhibits production of nitric oxide, pro-inflammatory cytokines, and inflammation-related proteins including p-STAT3. Additionally, bvPLA2 inhibits the production of Aβ in cultured BV-2 cells. Results from the docking experiment, pull-down assay, and the luciferase assay show that bvPLA2 directly binds STAT3 and, thus, regulates gene expression levels. Moreover, when the STAT3 inhibitor and bvPLA2 were administered together, the anti-amyloidogenic and anti-inflammatory effects were further enhanced than when they were administered alone.

**Conclusion:**

These results suggest that bvPLA2 could restore memory by inhibiting the accumulation of Aβ and inflammatory responses via blockage of STAT3 activity.

**Electronic supplementary material:**

The online version of this article (10.1186/s40035-019-0167-7) contains supplementary material, which is available to authorized users.

## Background

Alzheimer’s disease (AD) is the most common neurodegenerative disease that causes dementia and characteristically decreased cognitive function, including memory deterioration. In 2018, 5.7 million people in the United States were reported to suffer from AD, with 81% of them older than 75 years [1]. As the global proportion of people over 65 years of age continues to increase, the number of people suffering from AD is thought to increase as well [2]. Currently, there are six drugs approved by the United States Food and Drug Administration (FDA) for the treatment of AD: rivastigmine, galantamine, donepezil, memantine, memantine combined with donepezil, and tacrine. However, none of these treatments can stop or recover the damage and destruction of neurons that are thought to cause AD, and only temporarily relieve symptoms [1–3]. Therefore, there is a continuing need to develop drugs that can fundamentally treat AD.

On a cellular level, AD is characterized by the accumulation of extracellular plaques composed of polymerized amyloid beta (Aβ) peptides [4]. A major hypothesis explaining the pathogenesis of AD is the amyloid cascade hypothesis; the deposition of Aβ is a causative factor of AD, with neurofibrillary tangles, neuroinflammation, cell death, and dementia following as direct consequences [5, 6]. Although Aβ is mainly produced by neurons, other cell types, including astrocytes and other glial cells, produce Aβ under stress conditions that induce glial activation, such as occurs in AD [7]. Aβ is formed by sequential proteolysis of larger amyloid precursor proteins (APP). APP is first cleaved by β-secretase to produce C99, which is then cleaved by γ-secretase to produce Aβ [7, 8].

AD pathology also features an inflammatory response, which is mainly induced by intrinsic myeloid cells (microglia) and exacerbated by the progression of AD [9]. Microglia can bind to soluble Aβ oligomers and fibrils through receptors such as toll-like receptors (TLRs) [10]. When Aβ is bound to the TLR4 receptor on microglia, they are activated chronically. The chronically activated microglia release inflammatory chemokines and cytokines such as interleukin (IL)-1, IL-6, and tumor necrosis factor α (TNF-α) [11, 12]. Previously we have conducted studies on substances that alleviate AD through attenuating neuroinflammation. For example, bee venom, Antarctic krill oil, and K284–6111 have been found to regulate the nuclear factor (NF)-κB signaling pathway to alleviate neuroinflammation and are effective in AD [5, 8, 13, 14].

Signal transducer and activator of transcription-3 (STAT3) is a key mediator of intracellular signaling and regulates the expression of various genes involved in inflammatory responses [15]. When the STAT3 dimer is activated through progressive phosphorylation, STAT3 translocates to the nucleus, where it binds to consensus STAT3-binding sequences located in the promoters of genes that encode proinflammatory mediators, including cytokines, chemokines, and inflammatory enzymes [16, 17]. STAT3 also triggers neuronal inflammation by promoting microglial and astrocytic activation [18]. Studies have found that STAT3 knockout mice showed anti-depressive-like behavior and that p-STAT3 levels are highly elevated in APP/PS1 mice and is involved in neuronal apoptosis [19, 20]. STAT3 binds to the promotor of β-secretase 1 (BACE1), and there is a correlation between levels of p-STAT3 and BACE1 [21]. Additionally, one study found that the level of presenilin-1 increased when the level of p-STAT3 increased in the nucleus, which was highly related to AD [22]. Thus, it is suggested that the STAT3 signal is significant in mediating the transcriptional regulation of BACE1 and presenilin-1, contributing to the development of AD.

Bee venom is composed of various peptides, enzymes, and various other substances, including melittin and phospholipase A2 (PLA2), which are known to have anti-inflammatory and analgesic effects [23]. PLA2 is an enzyme that catalyzes the hydrolysis of the sn-2 fatty acyl bond of membrane phospholipids to generate free fatty acids and lysophospholipids [24, 25]. Studies have shown that bvPLA2 has utility as a treatment for Parkinson’s disease, atopic dermatitis, and asthma [25–27]. In addition, in our previous study, we found that bee venom exerts anti-cancer activity through the STAT3 signaling pathway in ovarian cancer cells [28]. One study also found that bee venom inhibits STAT3 activation in mast cell-mediated allergic inflammatory responses [29]. Moreover, our previous study found that bee venom offers neuroprotective effects in a lipopolysaccharide (LPS)-induced AD mouse model [8], and other researchers have also reported that bee venom has therapeutic effects against other neurological diseases such as Parkinson’s disease [30, 31]. However, its anti-neuroinflammatory and anti-amyloidogenic effects in a Tg2576 mouse model have not yet been studied. Therefore, we investigated the effect of bvPLA2 on a Tg2576 AD mouse model and its mechanisms in terms of amyloidogenesis and neuroinflammation.

## Methods

### Materials

The bvPLA2 was supplied from INISTst Co., LTD (Gyeonggi-do, Republic of Korea) and was dissolved in phosphate-buffered saline (PBS; final concentration of 1 mg/mL) and stored at − 20 °C until use. The LPS was purchased from Sigma (serotype 0111:B4; Sigma, St. Louis, MO, USA). The LPS (final concentration of 1 mg/mL) was dissolved in PBS, and aliquots were stored at − 20 °C until use. The Aβ_1–42_ was purchased from Tocris Bioscience (rat; Bristol, United Kingdom). The Aβ_1–42_ (final concentration of 200 μM) was dissolved in 0.1% Ammonia and were stored at − 70 °C until use.

### Animal and treatment

Twelve month old Tg2576 mice were maintained and handled in accordance with the humane animal care and use guidelines of the Ministry of Food and Drug Safety. Tg2576 mice harboring human APP695 with Swedish double mutation (hAPP; HuAPP695; K670 N/M671 L) were purchased from Taconic Farms (Germantown, NY, USA), and the strain was maintained in the animal laboratory at Chungbuk National University. Tg2576 mice were randomly divided into two groups with 10 mice in each group: the control group and the bvPLA2 (1 mg/kg) -treated group. The bvPLA2 was administered intraperitoneally twice per week for 4 weeks. Control mice were alternatively given an equal volume of vehicle. The behavioral tests of learning and memory capacity were assessed using the water maze, probe, and passive avoidance tests. Mice were sacrificed after behavioral tests by CO_2_ asphyxiation.

### Morris water maze

The water maze test is a commonly accepted method for memory test, and we performed this test as described by Morris et al. [32]. Maze testing was carried out by the SMART-CS (Panlab, Barcelona, Spain) program and equipment. A circular plastic pool (height: 35 cm, diameter: 100 cm) was filled with water made opaque with skim milk kept at 22–25 °C. An escape platform (height: 14.5 cm, diameter: 4.5 cm) was submerged 1–1.5 cm below the surface of the water in position. Testing trials were performed on a single platform and at two rotational starting positions. After testing trial, the mice were allowed to remain on the platform for 120 s and were then returned to their cage. Escape latency and escape distance of each mouse was monitored by a camera above the center of the pool connected to a SMART-LD program (Panlab, Barcelona, Spain).

### Probe test

To assess memory retention, a probe test was performed 24 h after the water maze test. The platform was removed from the pool which was used in the water maze test, and the mice were allowed to swim freely. The swimming pattern of each mouse was monitored and recorded for 60 s using the SMART-LD program (Panlab, Barcelona, Spain). Retained spatial memory was estimated by the time spent in the target quadrant area.

### Passive avoidance performance test

The passive avoidance test is generally accepted as a simple method for testing memory. The passive avoidance response was determined using a “step-through” apparatus (Med Associates Inc., Vermont, USA) that is divided into an illuminated compartment and a dark compartment (each 20.3 × 15.9 × 21.3 cm) adjoining each other through a small gate with a grid floor, 3.175 mm stainless steel rods set 8 mm apart. On the first day, the mice were placed in the illuminated compartment facing away from the dark compartment for the training trial. When the mice moved completely into the dark compartment, it received an electric shock (0.45 mA, 3 s duration). Then the mice were returned to their cage. One day after training trial, the mice were placed in the illuminated compartment and the latency period to enter the dark compartment defined as “retention” was measured. The time when the mice entered into the dark compartment was recorded and described as step-through latency. The retention trials were set at a cutoff time limit of 3 min.

### Collection and preservation of brain tissues

After behavioral tests, mice were perfused with PBS with heparin under inhaled CO_2_ anesthetization. The brains were immediately removed from the skulls, after that, only the hippocampus region was isolated and stored at − 80 °C until biochemical analysis.

### Thioflavin S staining

After being transferred to 30% sucrose solutions, brains were cut into 20-μm sections by using a cryostat microtome (Leica CM 1850; Leica Microsystems, Seoul, Korea). After washes in distilled water for 5 min, brain sections were transferred to gelatin-coated slides and placed in 1% thioflavin S (Sigma, St Louis, MO, USA) in 50% ethanol for 8 min. Brain sections were then washed in distilled water and then dehydrated through ascending grades of ethanol, 50, 70, 90, and 100% ethanol for 2 min in each grade. The sections were then mounted in a mounting medium (Fluoromount™ Aqueous Mounting Medium; Sigma, St. Louis, MO, USA). The thioflavin S staining was examined using a fluorescence microscope (Axio Observer A1; Carl Zeiss, Oberkochen, Germany) (× 50 and × 200).

### Assay of β-secretase activities

β-secretase activity in the mice brains was determined using a commercially available β-secretase activity kit (Abcam, Inc., Cambridge, MA, USA). Solubilized membranes were extracted from brain tissues using β-secretase extraction buffer, incubated on ice for 1 h and centrifuged at 5000×g for 10 min at 4 °C. The supernatant was collected. A total of 50 μL of sample (total protein 100 μg) or blank (β-secretase extraction buffer 50 μL) was added to each well (used 96-well plate) followed by 50 μL of 2× reaction buffer and 2 μL of β-secretase substrate incubated in the dark at 37 °C for 1 h. Fluorescence was read at excitation and emission wavelengths of 335 and 495 nm, respectively, using a fluorescence spectrometer (Gemini EM; Molecular Devices, CA, USA).

### Measurement of Aβ

Lysates of brain tissue were obtained through a protein extraction buffer containing protease inhibitor. Aβ_1–42_ and Aβ_1–40_ levels were determined using each specific mouse amyloid beta peptide 1–42 enzyme-linked immunosorbent assay (ELISA) Kit (CSB-E10787m; CUSABIO, Houston, USA) and mouse amyloid beta peptide 1–40 ELISA Kit (CSB-E08300m; CUSABIO, Houston, USA). Protein was extracted from brain tissues using a protein extraction buffer (PRO-PREP; Intron Biotechnology, Kyungki-do, Korea), incubated on ice for 1 h, and centrifuged at 13,000×g for 15 min at 4 °C. In brief, 100 μL of sample was added into a precoated plate and incubated for 2 h at 37 °C. After removing any unbound substances, a biotin-conjugated antibody specific for Aβ was added to the wells. After washing, avidin-conjugated horseradish peroxidase (HRP) was then added to the wells. Following a wash to remove any unbound avidin-enzyme reagent, a substrate solution was added to the wells and color developed in proportion to the amount of Aβ bound in the initial step. The color development was stopped and the intensity of the color was measured.

### Immunohistochemical staining

After being transferred to 30% sucrose solutions, brains were cut into 20-μm sections using a cryostat microtome (Leica CM 1850; Leica Microsystems, Seoul, Korea). After two washes in PBS (pH 7.4) for 10 min each, the samples were incubated with 3% hydrogen peroxide and 1% Triton-X in PBS for 30 min for antigen retrieval and blocking of endogenous peroxidase, followed by an additional two washes in PBS for 10 min each. The brain sections were blocked for 1 h in 3% bovine serum albumin (BSA) solution and incubated overnight at 4 °C with glial fibrillary acidic protein (GFAP; 1:300; Santa Cruz Biotechnology, Inc., Santa Cruz, CA, USA), inducible nitric oxide synthase (iNOS; 1:300; Abcam, Inc., Cambridge, MA, USA), ionized calcium binding adaptor molecule 1 (IBA-1; 1:300; Abcam, Inc., Cambridge, MA, USA), and cyclooxygenase 2 (COX-2; 1:300, Novus Biologicals, Inc., CO, USA). After incubation with the primary antibodies, brain sections were washed three times in PBS for 10 min each. After washing, brain sections were incubated for 1–2 h at room temperature with the biotinylated goat anti-rabbit, goat anti-mouse, or donkey anti-goat IgG-horseradish peroxidase (HRP) secondary antibodies (1:500; Santa Cruz Biotechnology, Inc., Santa Cruz, CA, USA). Brain sections were washed three times in PBS for 10 min each and visualized by a chromogen diaminobenzidine (Vector Laboratories, Burlingame, CA, USA) reaction for up to 10 min. Finally, brain sections were dehydrated in ethanol, cleared in xylene, mounted with Permount (Fisher Scientific, Hampton, NH, USA), and evaluated on a light microscope (Microscope Axio Imager. A2; Carl Zeiss, Oberkochen, Germany; × 50 and × 200).

### Western blot analysis

Western blotting was performed as described [33]. To detect target proteins, specific antibodies against iNOS, IBA-1, GFAP, APP, and BACE1 (1:1000; Abcam, Inc., Cambridge, UK), COX-2 (1:1000; Novus Biologicals, Inc., CO, USA), c-Jun N-terminal kinase (JNK), extracellular signal–regulated kinase (ERK) 1/2, p-p38 and p38(1:000; Cell signaling Technology, Inc., MA, USA), p-STAT3, STAT3, p-ERK1/2, p-JNK, and β-actin (1:200; Santa Cruz Biotechnology Inc., Santa Cruz, CA, USA) were used. The blots were then incubated with the corresponding conjugated secondary antibodies such as anti-mouse, anti-rabbit and anti-goat purchased from Santa Cruz Biotechnology Inc. (Santa Cruz, CA, USA). Immunoreactive proteins were detected with an enhanced chemiluminescence Western blotting detection system.

### Measurement of cytokines level

The pro-inflammatory and anti-inflammatory cytokines level was measured by quantitative reverse transcription polymerase chain reaction (qRT-PCR). Total RNA was extracted using RiboEX (Geneall biotechnology, Seoul, Korea) from hippocampus tissue and cDNA was synthesized using High-Capacity cDNA Reverse Transcription kit (Thermo Scientific, Waltham, MA, USA). Quantitative real-time PCR was performed on a 7500 real-time PCR system (Applied Biosystems, Foster City, CA, USA) for custom-designed primers and β-actin was used for house-keeping control using HiPi Real-Time PCR SYBR green master mix (ELPIS biotech, Daejeon, Korea). Cycling conditions consisted of a initial denaturation step of 3 min at 94 °C, a denaturation step of 30 s at 94 °C, an annealing step of 30 s at 60 °C and a extension step of a minute at 72 °C followed by 40 cycles. The values obtained for the target gene expression were normalized to β-actin and quantified relative to the expression in control samples.

Each sample was run with the following primer pairs: β-actin, Forward primer: 5′- GGCTGTATTCCCCTCCATCG-3′, Reverse primer: 5′- CCAGTTGGTAACAATGCCATGT-3′; TNF-α, Forward primer: 5′-TCTTCTCATTCCTGCTTGTGG-3′, Reverse primer: 5′- CACTTGGTGGTTTGCTACGA-3′; IL-1β, Forward primer: 5′-CCTTCCAGGATGAGGACATGA-3′, Reverse primer: 5′-TGAGTCACAGAGGATGGGCTC-3′; IL-6, Forward primer: 5′-GAGGATACCACTCCCAACAGACC-3′, Reverse primer: 5′-AAGTGCATCATCGTTGTTCATACA-3′; IL-10, Forward primer: 5′-TCTGAGCCACTCACATCTGC-3′, Reverse primer: 5′-TCAGGGGAACTGCTAGTGCT-3′; IL-4, Forward primer: 5′-GGTCTCAACCCCCAGCTAGT-3′, Reverse primer: 5′-GCCGATGATCTCTCTCAAGTGAT-3′; TGF-β, Forward primer: 5′-CTCCCGTGGCTTCTAGTGC-3′, Reverse primer: 5′- GCCTTAGTTTGGACAGGATCTG -3′.

### BV-2 microglial cells culture

Microglial BV-2 cell cultures were performed as previously described [34]. The cultured cells were treated simultaneously with LPS (1 μg/mL) or Aβ_1–42_ (2.5 μM) and with several concentrations (0.01, 0.1, 1 μg/mL) of bvPLA2 dissolved in PBS. The cells were harvested after 24 h. Cell viability, Nitric Oxide concentration and level of proinflammatory cytokines were determined.

### Cell viability assay

BV-2 cells were plated in 96-well plates, subsequently treated with bvPLA2 (0–1 μg/mL) for 24 h. After treatment, cell viability was measured by MTT [3-(4, 5-Dimethylthiazol-2-yl)-2, 5-Diphenyltetrazolium Bromide] solution (Sigma Aldrich, St. Louis, MO). MTT solution having a volume of 1/10 of the culture medium was added to each well, and the mixture was incubated for 2 h in CO_2_ incubator and then removed. After removing the mixture from the cells, DMSO as much as the volume of the medium was added. Then the absorbance of each well was read at a wavelength of 570 nm using a microplate absorbance reader.

### Nitric oxide assay

BV-2 cells were grown in 96-well plates and then incubated with or without LPS (1 μg/mL) in the absence or presence of various concentrations of bvPLA2 (0.01, 0.1, 1 μg/mL) for 24 h. The nitrite concentration in the supernatant was assessed by NO detection kit (Intron Biotechnology, Kyungki-do, Korea). The absorbance at 520 nm was measured in a microplate absorbance reader, and a series of known concentrations of sodium nitrite was used as a standard.

### Pull-down assay

bvPLA2 was conjugated with Epoxy-activated Sepharose 4B (GE Healthcare Korea, Seoul, Korea). Briefly, bvPLA2 (1 mg) was dissolved in 1 mL of coupling buffer (0.1 M NaHCO_3_ and 0.5 M NaCl, pH 11.0). The Epoxy-activated Sepharose 4B beads (0.1 g) were swelled and washed in 1 mM HCl on a sintered glass filter, then washed with the coupling buffer. Epoxy-activated Sepharose 4B beads were added to the bvPLA2-containing coupling buffer and rotated at 4 °C overnight. The control unconjugated Sepharose 4B beads were prepared as described above in the absence of bvPLA2. After washing, unoccupied binding sites were blocked with a blocking buffer (0.1 M Tris-HCl, pH 8.0) at room temperature for 3 h. The bvPLA2-conjugated Sepharose 4B was washed with three cycles of alternating pH wash buffers (buffer 1: 0.1 M acetate and 0.5 M NaCl, pH 4.0; buffer 2: 0.1 M Tris-HCl and 0.5 M NaCl, pH 8.0). bvPLA2-conjugated beads were then equilibrated with a binding buffer (0.05 M Tris-HCl and 0.15 M NaCl, pH 7.5). To demonstrate binding of bvPLA2 and STAT3, the STAT3 protein was overexpressed by transfection with STAT3 DNA. BV-2 cells were transfected with STAT3 DNA (700 ng/per well of a six well plate) using Lipofectamine® 3000 (Invitrogen, Waltham, MA, USA) in Opti-MEM, following the manufacturer’s protocol. The BV-2 cell lysate (1 mg of protein) was mixed with bvPLA2-conjugated Sepharose 4B or unconjugated Sepharose 4B and incubated at 4 °C overnight. The beads were then washed three times with TBST. The bound proteins were eluted with sodium dodecyl sulfate (SDS) loading buffer and were separated using SDS/polyacrylamide gel electrophoresis, followed by immunoblotting with antibodies against STAT3 (1:200, Santa Cruz Biotechnology, Dallas, TX, USA).

### Luciferase assay

BV-2 cells were plated in 12-well plates (8 × 10^4^ cells/well) and transiently transfected with STAT3-Luc (Stratagene, La Jolla, CA, USA) plasmid, using Lipofectamine 3000 in Opti-MEM according to the manufacturer’s specifications (Invitrogen, Carlsbad, CA, USA) for 24 h. Subsequently, the transfected cells were treated with 1 μg/mL of LPS and 0.01, 0.1, and 1 μg/mL of bvPLA2 for 24 h. Luciferase activity was measured using a Dual-Luciferase® Reporter Assay System kit (Promega, Madison, WI, USA) and a luminometer according to the manufacturer’s specifications (WinGlow, Bad Wildbad, Germany).

### Docking experiment

A docking study of bvPLA2 with STAT3 was performed using Autodock VINA (Trott and Olson, 2010). Three-dimensional structures of the STAT3 [PDB: 3CWG] and bvPLA2 [PDB: 1POC] were retrieved from the Protein Data Bank, which was further prepared using AutodockTools. The grid box was centered on the STAT3 monomer, and the size of the grid box was adjusted to include the whole monomer. Docking experiments were performed at various default exhaustiveness values: 16, 24, 32, 40, and 60. Molecular graphics for the best binding model were generated using the Discovery Studio Visualizer 2.0.

### Statistical analysis

The data were analyzed using the GraphPad Prism software (Version 4.03; GraphPad software, Inc., San Diego, CA, USA). Data are presented as mean ± S.E.M. The differences in all data were assessed by one-way analysis of variance (ANOVA). When the *P* value in the student’s t-test indicated statistical significance, the differences were assessed by the Dunnett’s test. A value of *p* < 0.05 was considered to be statistically significant.

## Results

### bvPLA2 alleviates genetically induced memory impairments in Tg2576 mice

In the present study, we investigated Tg2576 mice, which are widely used as an AD mouse model. Tg2576 mice overexpress human APP with the Swedish mutation found in a familial form of AD in Sweden [35]. Tg2576 mice rapidly produce Aβ until up to 6 months of age and then begin to produce Aβ plaques between 9 and 12 months of age. At 13 months, the level of Aβ plaques rapidly accumulates and the mice display symptoms of AD, such as impaired cognitive abilities [36].

**Fig. 1:**
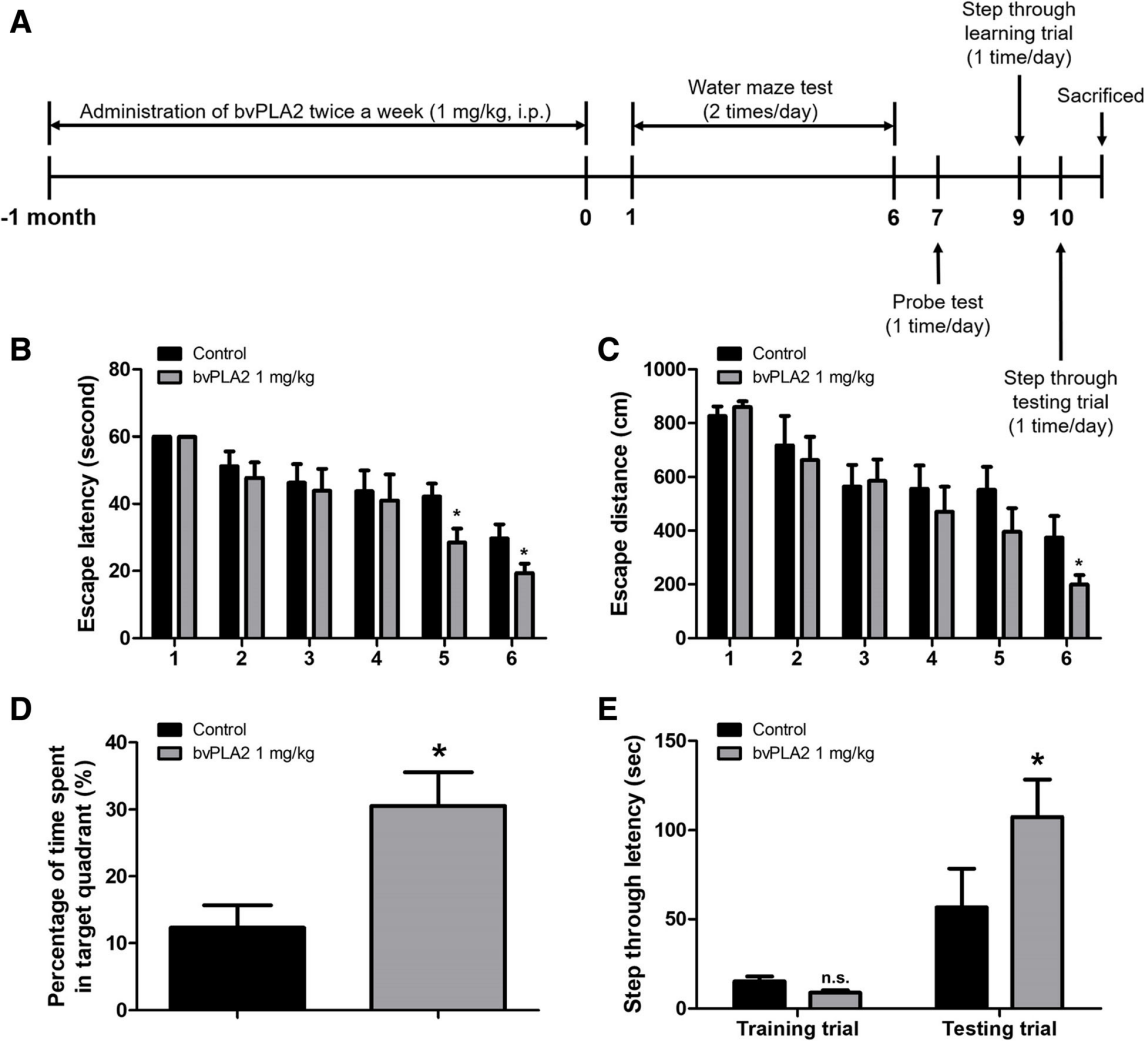
Effect of bvPLA2 on genetically induced memory impairment in Tg2576 mice. A timeline have been described that demonstrate the administration of bvPLA2 and the assessment of cognitive function in Tg2576 mice (**a**). To investigate effect of bvPLA2 on memory impairment, we carried out the water maze test (**b**, **c**), the probe test (**d**), and the step-through type passive avoidance test (**e**). Memory and learning ability in Tg2576 were determined by the escape latencies (**b**, sec) and escape distance (**c**, cm) for 6 days, and time spent in target quadrant (**d**, %) in the probe test. Each value is mean ± S.E.M. from 10 mice. *, Significantly different from control group (*p* < 0.05)

To investigate the memory improvement effect of bvPLA2 in the Tg2576 AD mouse model, mice received an intraperitoneal injection of bvPLA2 (1 mg/kg) twice per week for 4 weeks (Fig. 1a). After 4 weeks of administration, Morris Water Maze, probe, and passive avoidance tests were performed serially to assess spatial learning ability and memory. Escape latency and distance were measured to determine the effect of bvPLA2 on memory improvements (Fig. 1b, c). The mean escape latency and swimming distance of the control group on day 6 were approximately 29.71 ± 4.189 s and 346.1 ± 71.43 cm, respectively. Alternatively, the mean escape latency and swimming distance of the bvPLA2-treated group were 19.36 ± 2.790 s and 186.8 ± 28.48 cm, respectively, demonstrating a significantly decreased average escape latency and distance compared to those in the control group. The retention ability of memory was measured by the probe test the day after the final day of water maze testing (Fig. 1d). The mean time to stay in the target quadrant increased significantly in the bvPLA2-treated group (30.54 ± 4.992%) compared to that in the control group (12.31 ± 3.355%). The passive avoidance test was performed 2 days after the probe test (Fig. 1e). The control group showed an average step through latency of 36.17 ± 8.310 s in the illuminated compartment, but the average step-through latency of the bvPLA2-treated group was 107.5 ± 20.90 s, which was significantly higher than that of the control group.

### Inhibitory effect of bvPLA2 on accumulation of Aβ

**Fig. 2:**
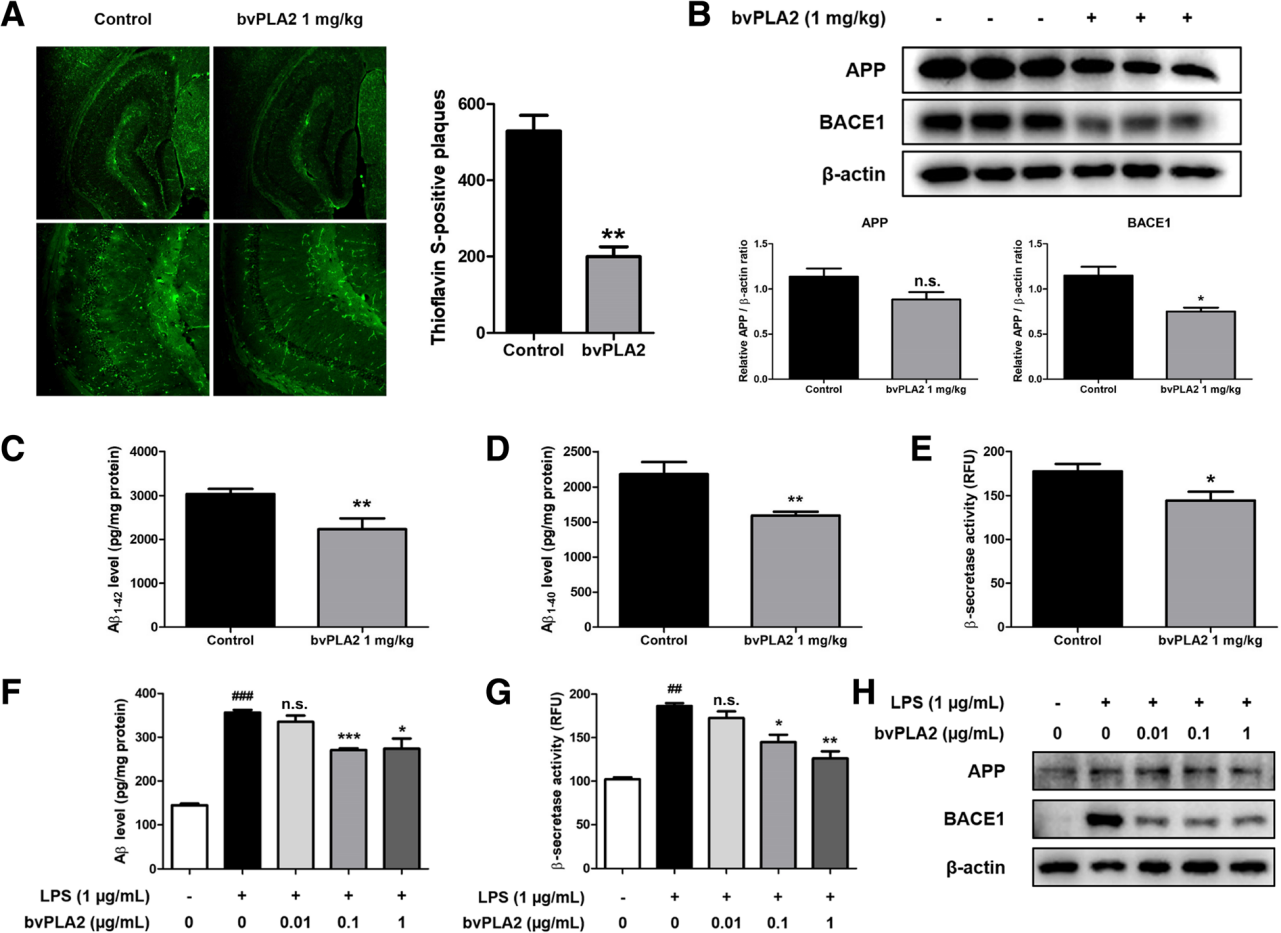
Inhibitory effect of bvPLA2 on accumulation of Aβ and factors involved in Aβ production. The accumulation of Aβ plaques in Tg2576 mice brain was determined by Thioflavin S staining (**a**). The expression of APP and BACE1 were detected by Western blotting using specific antibodies in the mice brain (**b**). Each blot is representative for three experiments. For the cropped images, samples were run in the same gels under the same experimental conditions and processed in parallel. Band density was quantified from three mice (**b**). The levels of Aβ_1–42_ (**c**) and Aβ_1–40_ (**d**) in Tg2576 mice brain were assessed by ELISA. The activity of β-secretase in mice brain was investigated by β-secretase activity assay kit (**e**). Values measured from each group of mice were calibrated by amount of protein and expressed as mean ± S.E.M. (*n* = 10). *, Significantly different from control group (*p* < 0.05). **, Significantly different from control group (*p* < 0.01). The levels of Aβ_1–42_ in BV-2 microglial cells were assessed by ELISA (**f**). The activity of β-secretase in BV-2 cells was investigated by β-secretase activity assay kit (**g**). Values measured from each group were calibrated by amount of protein and expressed as mean ± S.E.M. (*n* = 3). The expression of APP and BACE1 in BV-2 cells were detected by western blotting using specific antibodies (**h**). For the cropped images, samples were run in the same gels under the same experimental conditions and processed in parallel. ##, Significantly different from control group (p < 0.01). ###, Significantly different from control group (*p* < 0.001). *, Significantly different from LPS group (*p* < 0.05). **, Significantly different from LPS group (*p* < 0.01). ***, Significantly different from LPS group (*p* < 0.001)

Aβ accumulation is considered to be the most well-known causative factor for AD. Thus, we performed Thioflavin S staining to determine if bvPLA2 affects genetically induced Aβ accumulation in the brains of Tg2576 mice. Thioflavin S staining is used to stain β-sheet-rich structures of Aβ, which are mainly found on Aβ plaques. There was substantial decrease in the accumulation of Aβ plaques in the bvPLA2-treated group compared to that in the control group (Fig. 2a). The levels of APP and BACE1 involved in Aβ production were detected by using Western blot analysis in Tg2576 mice brains. Expression of APP and BACE1 levels were decreased in the brains of bvPLA2 administered mice compared to the control group (Fig. 2b). ELISA was performed to quantitatively measure Aβ_1–42_ levels and Aβ_1–40_ levels in Tg2576 mice brains. The Aβ_1–42_ level in the control group was 3039 ± 116.8 pg/mg of protein in the brain and 2236 ± 244.9 pg/mg of protein in the brain in the bvPLA2-treated group (Fig. 2c). The Aβ_1–40_ level in the control group was 2182 ± 168.6 pg/mg of protein in the brain and 1593 ± 53.25 pg/mg of protein in the brain in the bvPLA2-treated group (Fig. 2d). Comparing these amyloid levels between the two groups, administration of bvPLA2 significantly reduced Aβ levels in the brains of Tg2576 mice. To determine how amyloid production was reduced by bvPLA2, we analyzed β-secretase activity in the brain. We found that β-secretase activity significantly decreased with administration of bvPLA2 (Fig. 2e).

The effect of bvPLA2 on cell viability in BV-2 cells was first determined by MTT assay (Fig. 4a). There was no significant change in cell viability in the bvPLA2 (0.01, 0.1, 1 μg/mL) -treated groups compared to that in the control group. Thus, bvPLA2 does not appear to be toxic to BV-2 cells up to 1 μg/mL. To investigate the effects of bvPLA2 on amyloidogenesis in vitro, microglial BV-2 cells were treated with LPS and several concentrations of bvPLA2 (0.01, 0.1, 1 μg/mL) for 24 h. The Aβ_1–42_ levels in BV-2 cells were determined by ELISA. Aβ_1–42_ levels in the control group were 144.9 ± 3.587 pg/mg of protein and in the LPS group, they were significantly increased (356.5 ± 6.030 pg/mg protein; Fig. 2f). In the bvPLA2-treated groups, however, the levels were decreased in a concentration dependent manner and decreased up to 273.7 ± 23.33 pg/mg protein (bvPLA2 1 μg/mL-treated group). β-secretase activity was also significantly increased in the LPS group compared to that in the control group, but decreased in a concentration-dependent manner with bvPLA2 treatment (Fig. 2g). The expression levels of APP and BACE1 were measured by Western blot analysis (Fig. 2h). Expression of APP and BACE1 were significantly increased in the LPS group compared to that in the control group; however, the elevated expression level was decreased in the bvPLA2-treated groups.

### Effect of bvPLA2 on neuroinflammation in Tg2576 mice brains

**Fig. 3:**
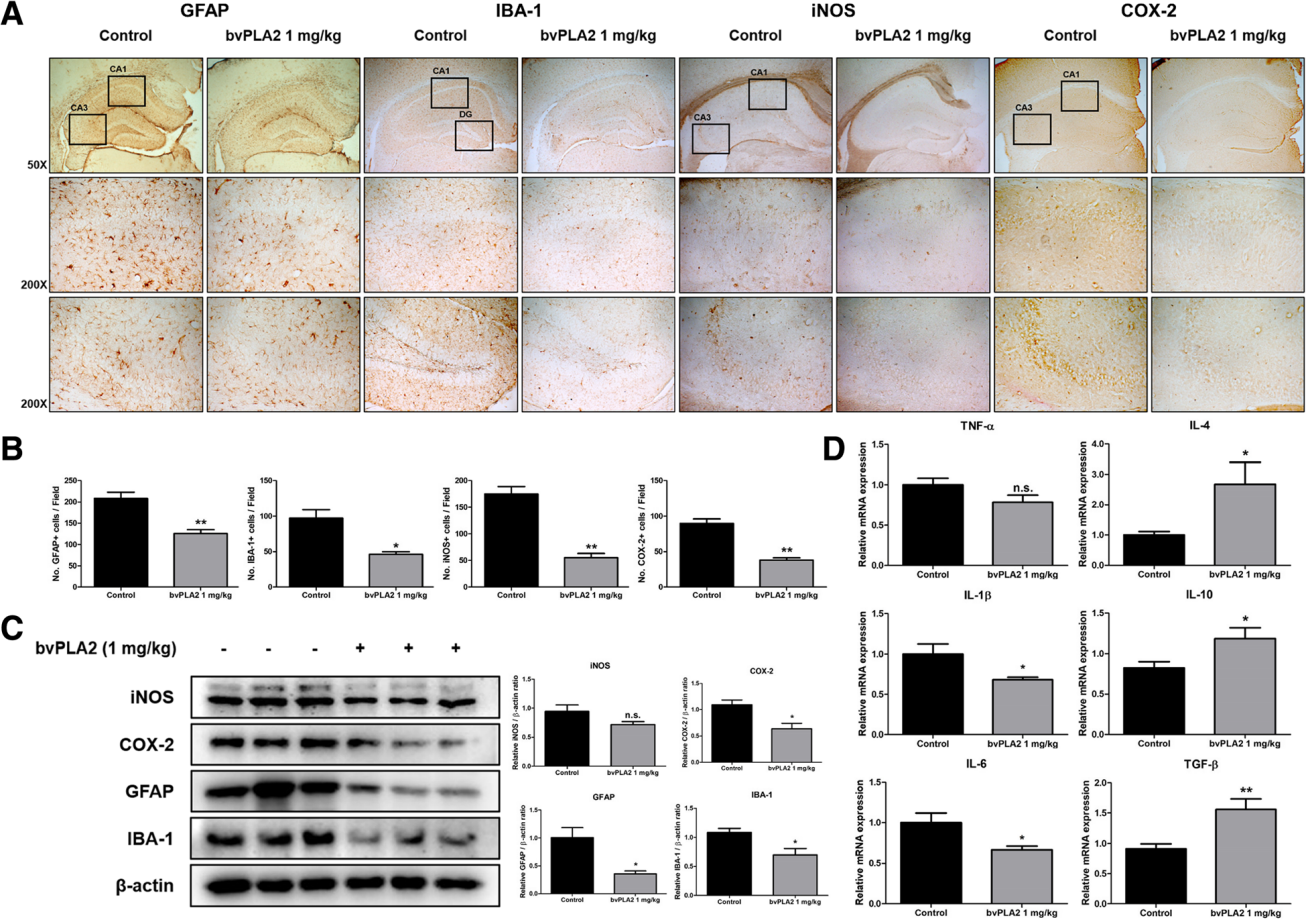
Inhibitory effect of bvPLA2 on genetically induced neuroinflammation in Tg2576 mice. Immunostaining of GFAP, IBA-1, iNOS, and COX-2 in the hippocampus were performed in 20 μm-thick sections of mice brain with specific primary antibodies and the biotinylated secondary antibodies (**a**). GFAP, IBA-1, iNOS, and COX-2 stainings were quantified by counting the number of positive cells in the field (**b**). The expression of iNOS, COX-2, GFAP and IBA-1 were detected by Western blotting using specific antibodies in the mice brain (**c**). Each blot is representative for three experiments. For the cropped images, samples were run in the same gels under the same experimental conditions and processed in parallel. Band density was quantified from three mice (**c**). The mRNA expression level of pro-inflammatory cytokines (TNF-α, IL-1β, and IL-6) and anti-inflammatory cytokines (IL-4, IL-10, and TGF-β) in the mice brain hippocampus site were assessed by qRT-PCR (**d**). Each value is mean ± S.E.M. from 10 mice. *, Significantly different from control group (*p* < 0.05). **, Significantly different from control group (*p* < 0.01)

Another feature of AD is the activation of astrocytes and microglia cells (i.e., neuroinflammation). The immunohistochemistry and Western blotting was carried out to investigate the expression levels of neuroinflammation-related proteins in the mouse brain. The number of GFAP and IBA-1, the markers of reactive astrocyte and activated microglia, respectively, were reduced in the bvPLA2-treated mice compared to that in the control group (Fig. 3a, b). The number of iNOS and COX-2, accepted inflammatory proteins, were also reduced in the bvPLA2-treated group (Fig. 3a, b). Expression levels of iNOS and COX-2 were reduced in the Tg2576 mice brains by administration of bvPLA2. The expression levels of GFAP and IBA-1 were also significantly decreased in the brains of the bvPLA2-treated group (Fig. 3c). The production of pro-inflammatory cytokines is well known as a marker the generation of inflammation, and the production of anti-inflammatory cytokines is indicative of a diminution of inflammation. qRT-PCR was performed to determine the level of production of proinflammatory cytokines and anti-inflammatory cytokines in the mice brains (Fig. 3d). The mRNA levels of pro-inflammatory cytokines such as TNF-α, IL-1β, and IL-6 in the brains of the bvPLA2-treated group were reduced compared to those in the control group. Alternatively, mRNA levels of anti-inflammatory cytokines such as IL-4 and TGF-β were significantly increased in the bvPLA2-treated group compared to those in the control group. However, the expression level of IL-10, an anti-inflammatory cytokine, was decreased in bvPLA2-treated mice brains.

### Effect of bvPLA2 on neuroinflammation in microglial BV-2 cells

**Fig. 4:**
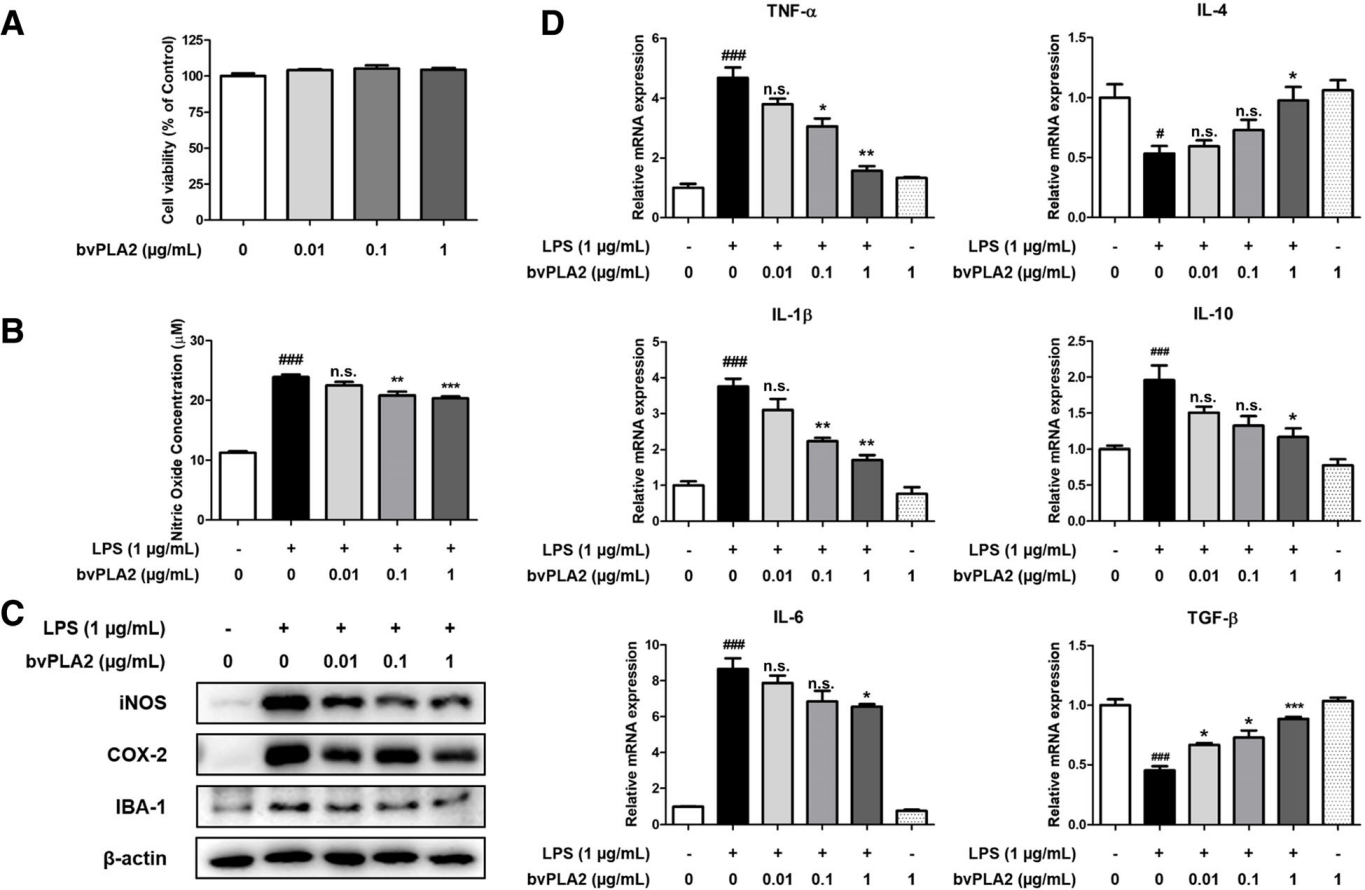
Inhibitory effect of bvPLA2 on LPS-induced neuroinflammation in BV-2 cells. The cell viability in BV-2 cells by bvPLA2 were determined by MTT assay (**a**). The effect of bvPLA2 on Nitric Oxide production in BV-2 cells were measured by Nitric Oxide assay (**b**). The expression of iNOS, COX-2, and IBA-1 were detected by Western blotting using specific antibodies in BV-2 cells treated with bvPLA2 (**c**). β-actin was used as a loading control. For the cropped images, samples were run in the same gels under same experimental conditions and processed in parallel. The mRNA expression level of pro-inflammatory cytokines (TNF-α, IL-1β, and IL-6) and anti-inflammatory cytokines (IL-4, IL-10, and TGF-β) in BV-2 cells treated with bvPLA2 were assessed by qRT-PCR (**d**). Each data representative for three different experiments. Each value is mean ± S.E.M. from 3 samples. #, Significantly different from control group (*p* < 0.05). ###, Significantly different from control group (*p* < 0.001). *, Significantly different from LPS group (*p* < 0.05). **, Significantly different from LPS group (*p* < 0.01). ***, Significantly different from LPS group (*p* < 0.001)

Anti-inflammatory effects of bvPLA2 in microglia cells were investigated in terms of nitric oxide concentration and expression levels of inflammatory-related proteins, pro-inflammatory cytokines, and anti-inflammatory cytokines. Nitric oxide concentration was significantly decreased up to 14.92 ± 2.154% in the bvPLA2-treated group compared to that in the LPS-treated group (Fig. 4b). The expression of iNOS, COX-2, and IBA-1 was also significantly decreased in the bvPLA2-treated groups in a concentration-dependent manner compared to that in the LPS-treated group (Fig. 4c). Levels of pro-inflammatory cytokines such as TNF-α, IL-1β, and IL-6 were significantly increased by LPS and decreased in a concentration-dependent manner in bvPLA2-treated groups (Fig. 4d). Alternatively, the expression level of anti-inflammatory cytokines such as IL-4 and TGF-β was significantly decreased by LPS treatment but recovered in a concentration-dependent manner by bvPLA2 treatment. However, the level of IL-10 was increased in the LPS group and decreased in the bvPLA2 (0.01, 0.1, 1 μg/mL) -treated groups in vivo. We further performed Western blot analysis and qRT-PCR as to whether bvPLA2 has an inhibitory effect on Aβ-induced neuroinflammation (Additional file 1: Figure S1). The expression of iNOS, COX-2, and p-STAT3 was significantly decreased in the bvPLA2-treated groups in a concentration-dependent manner compared to that in the Aβ-treated group (Additional file 1: Figure S1A, B). Levels of pro-inflammatory cytokines (TNF-α, IL-1β, and IL-6) were significantly increased by Aβ treatment and decreased in a concentration-dependent manner in bvPLA2-treated groups (Additional file 1: Figure S1C). These results suggest that bvPLA2 has inhibitory effects on the inflammatory response induced by LPS or Aβ in microglial BV-2 cells.

### Inhibitory effect of bvPLA2 on STAT3 activation through binding of bvPLA2 and STAT3

**Fig. 5:**
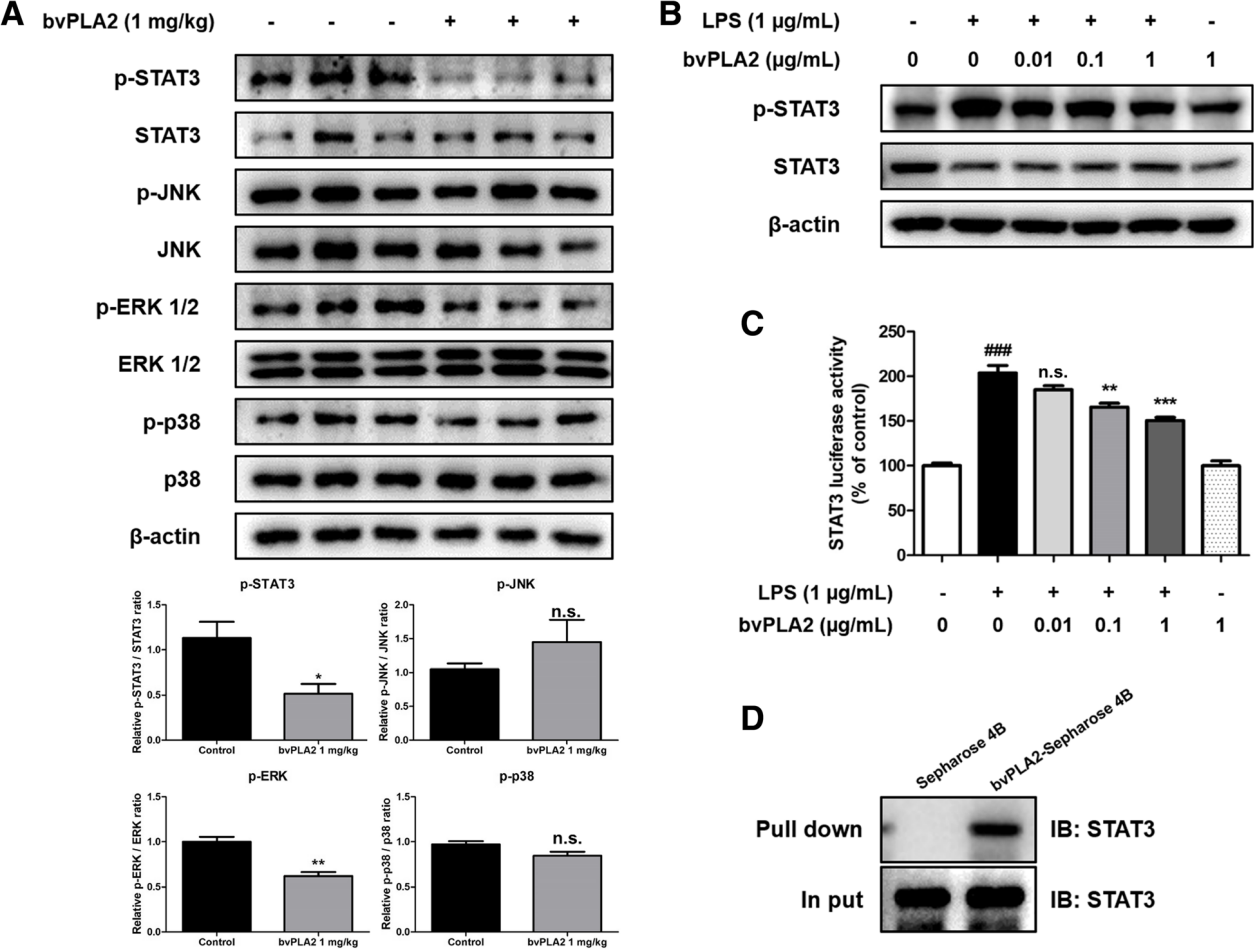
Inhibitory effect of bvPLA2 on STAT3 activation. The expression of p-STAT3, STAT3, p-JNK, JNK, p-ERK, ERK, p-p38 and p38 were detected by Western blotting using specific antibodies in the mice brain (**a**). Each blot is representative for three experiments. For the cropped images, samples were run in the same gels under the same experimental conditions and processed in parallel. Band density was quantified from three mice. *, Significantly different from control group (*p* < 0.05). **, Significantly different from control group (*p* < 0.01). The expression of p-STAT3 and STAT3 in BV-2 cells were detected by western blotting using specific antibodies (**b**). For the cropped images, samples were run in the same gels under the same experimental conditions and processed in parallel. Luciferase assay was carried out to determine the effects of bvPLA2 on LPS-induced STAT3 luciferase activity (**c**). Each data representative for three different experiments. Each value is mean ± S.E.M. from 3 samples. Whole cell lysate of BV-2 cells were incubated with bvPLA2-conjugated Sepharose 4B. After precipitation, the levels of bound STAT3 were monitored by Western blot analysis (**d**)

To investigate which signaling pathway was involved in the anti-neuroinflammatory effects of bvPLA2, the levels of factors involved in STAT3 and mitogen-activated protein kinases (MAPK) signaling pathway, which are highly related to inflammation, were studied using Western blotting in Tg2576 mice brains. The level of p-STAT3 was significantly decreased, and only the level of p-ERK in the MAPK signals was significantly decreased in the bvPLA2-treated group (Fig. 5a). bvPLA2 was thought to be closely related to the STAT3 signaling pathway; therefore, the level of p-STAT3 was examined by treating BV-2 cells activated by LPS (1 μg/mL) with bvPLA2 (0.01, 0.1, 1 μg/mL). In the Western blot analysis, the level of p-STAT3 was significantly increased by LPS treatment compared to that in the control but decreased by bvPLA2 treatment in a concentration-dependent manner. When bvPLA2 was treated at 1 μg/mL, the level of p-STAT3 was decreased to a level similar to that of the control (Fig. 5b). A luciferase activity assay was also carried out to investigate the effect of bvPLA2 on translational activity for the STAT3 promotor. We found that bvPLA2 reduced STAT3 luciferase activity in a concentration dependent manner in BV-2 cells (Fig. 5c). To determine if the effect of bvPLA2 could be associated with binding to STAT3, we performed a pull-down assay and a docking experiment between bvPLA2 and STAT3. We incubated whole cell lysate from BV-2 cells overexpressing STAT3 in Sepharose 4B beads and bvPLA2-conjugated Sepharose 4B beads and then detected by immunoblotting with anti-STAT3 antibody. STAT3 protein level was higher in bvPLA2-Sepharose 4B beads, suggesting that bvPLA2 could directly bind to STAT3 (Fig. 5d).

### bvPLA2 directly binds to LD of STAT3

**Fig. 6:**
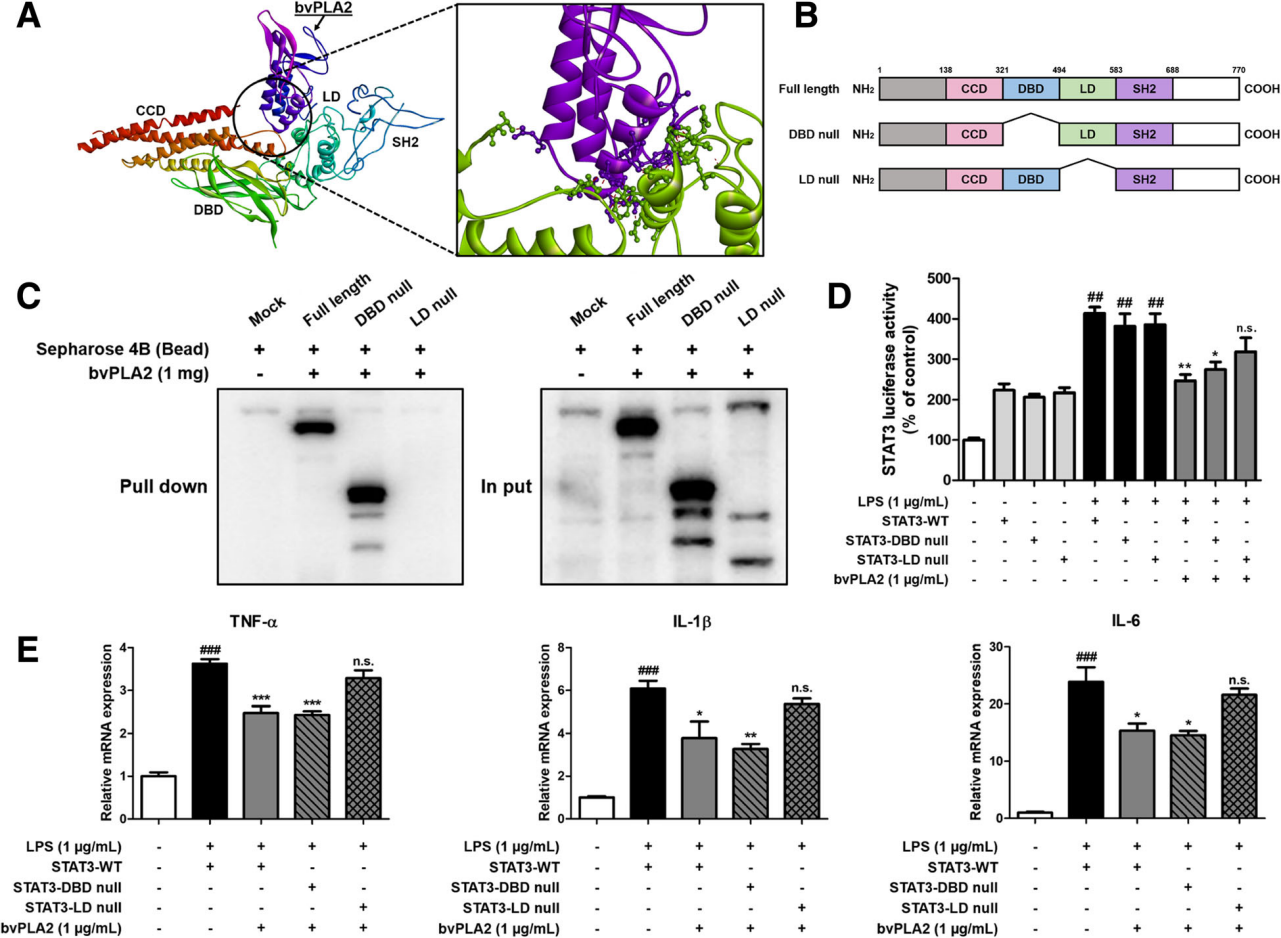
bvPLA2 directly binds to LD of STAT3. Docking model of bvPLA2 and STAT3 (**a**). In the enlarged image, purple part means bvPLA2 and green part means STAT3 (**a**). Schematic domain structures of several forms of STAT3 recombinant proteins (**b**). Pull-down assay with deletion of binding domains of STAT3 was performed to determine whether bvPLA2 binds to LD of STAT3 (**c**). Luciferase assay was performed to investigate the effects of bvPLA2 on LD-null STAT3 on LPS-induced STAT3 luciferase activity (**d**). The mRNA expression levels of pro-inflammatory cytokines (TNF-α, IL-1β, and IL-6) in BV-2 cells transfected with several mutant forms of STAT3 were assessed by qRT-PCR (**e**). Each data representative for three different experiments. Each value is mean ± S.E.M. from 3 samples. ##, Significantly different from control group (*p* < 0.01). ###, Significantly different from control group (*p* < 0.001). *, Significantly different from LPS-treated wild type group (*p* < 0.05). **, Significantly different from LPS-treated wild type group (*p* < 0.01). ***, Significantly different from LPS-treated wild type group (p < 0.001)

To identify the site where bvPLA2 binds to STAT3, we performed computational molecular docking modeling between bvPLA2 and STAT3 (Fig. 6a). The binding study showed that bvPLA2 binds directly to several amino acids in STAT3, especially to the linker domain (LD) of STAT3 (inside a binding pocket comprised of Thr500, Asp502, Gln503, Glu506, Ser509, Trp510, Ser513, Lys517, Arg518, Leu520, and Ile522). To elucidate which domain of STAT3 interacts with bvPLA2, we performed the pull-down assay, the luciferase activity assay and qRT-PCR with STAT3 in several mutant forms, such as wild type, DNA binding domain (DBD)-null and LD-null. (Fig. 6b). To confirm the interaction between bvPLA2 and the LD of STAT3, we performed the pull-down assay using bvPLA2-Sepharose 4B beads and cell lysates transfected with wild type (WT)-STAT3, DBD-null STAT3, and LD-null STAT3 (Fig. 6c). The bvPLA2-Sepahrose 4B beads pulled down the WT-STAT3 and DBD-null STAT3, whereas LD-null STAT3 could not. The luciferase activity of STAT3 was significantly increased by treatment of LPS in all of the microglial BV-2 cells transfected with WT-STAT3, DBD-null STAT3, or LD-null STAT3 (Fig. 6d). We found that bvPLA2 decreased the STAT3 luciferase activity in the BV-2 cells with WT-STAT3 vector or DBD-null STAT3 vector except with LD-null STAT3 vector. The qRT-PCR was carried out to investigate whether the anti-inflammatory effect of bvPLA2 differs in BV-2 cells transfected with WT-STAT3, DBD- null STAT3, or LD-null STAT3 (Fig. 6e). The levels of TNF-α, IL-1β, and IL-6 were decreased by bvPLA2 in the BV-2 cells transfected with the WT-STAT3 or DBD-null STAT3, but these cytokine levels were hardly affected by treatment of bvPLA2 in the BV2 cells transfected with the LD-null STAT3. These results indicated that bvPLA2 could suppress the activation of STAT3 by binding to the LD of STAT3.

### Synergistic effects of bvPLA2 and STAT3 inhibitor on amyloidogenesis and neuroinflammation

**Fig. 7:**
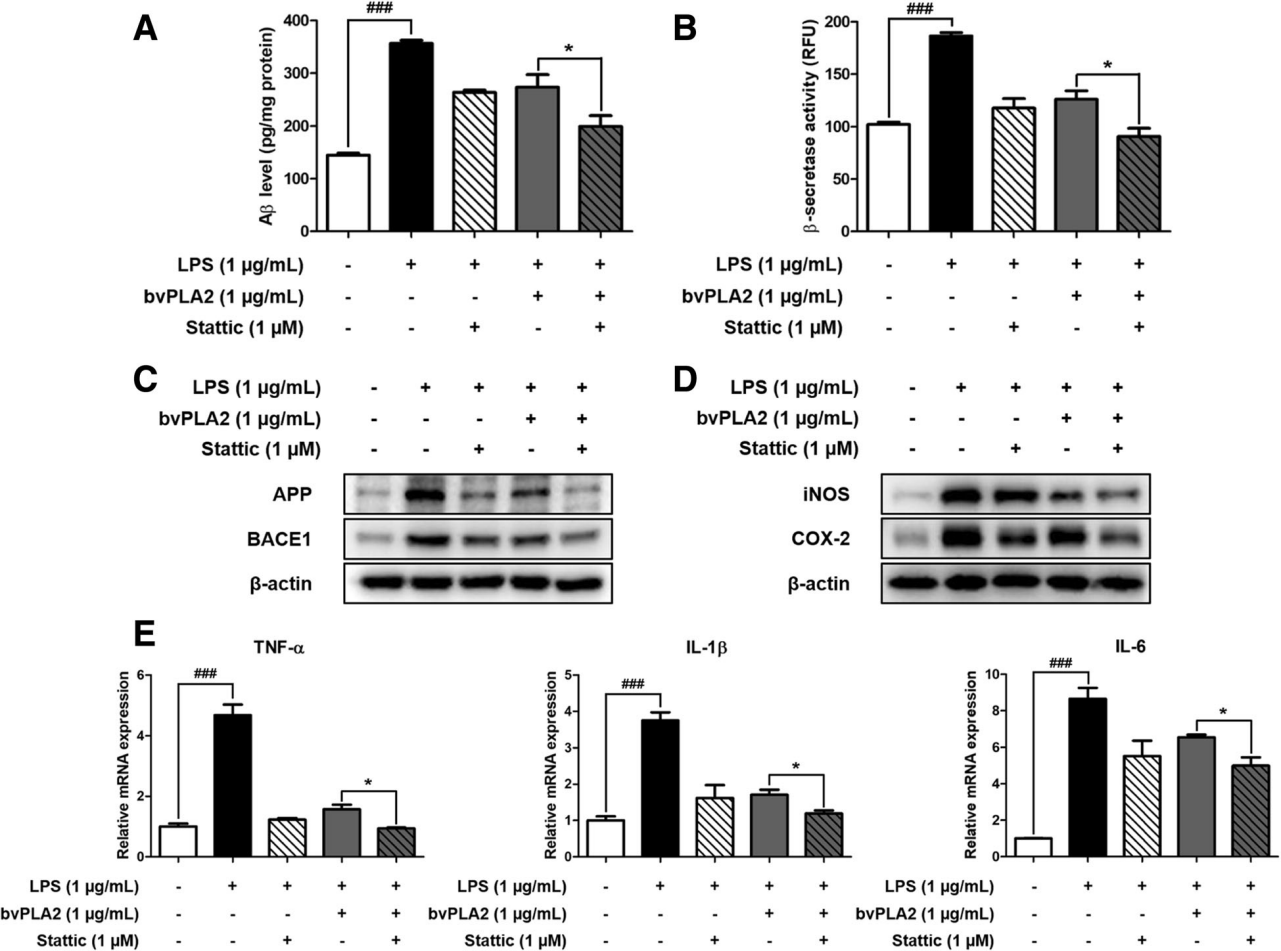
Combination effects of bvPLA2 and STAT3 inhibitor in BV-2 cells. The levels of Aβ_1–42_ in BV-2 cells were assessed by ELISA (**a**). The activity of β-secretase in BV-2 cells were investigated by β-secretase activity assay kit (**b**). The expression of APP and BACE1 in BV-2 cells were detected by Western blotting using specific antibodies (**c**). The expression of iNOS and COX-2 in BV-2 cells were detected by western blotting using specific antibodies (**d**). For the cropped images, samples were run in the same gels under the same experimental conditions and processed in parallel. The mRNA expression level of pro-inflammatory cytokines (TNF-α, IL-1β, and IL-6) in BV-2 cells treated with bvPLA2 or Stattic were assessed by qRT-PCR (**e**). Each data representative for three different experiments. Each value is mean ± S.E.M. from 3 samples. ###, Significantly different from control group (*p* < 0.001). *, Significantly different from bvPLA2 treated group (*p* < 0.05)

The inhibitory effects of STAT3 inhibitor (Stattic) and bvPLA2 on amyloidogenesis and neuroinflammation were tested in BV-2 cells. To determine the combination effects of bvPLA2 and Stattic on amyloidogenesis and neuroinflammation in vitro, microglial BV-2 cells were treated with LPS (1 μg/mL), Stattic (1 μM), or bvPLA2 (1 μg/mL) for 24 h. The Aβ_1–42_ levels in BV-2 cells were measured by ELISA (Fig. 7a). The Aβ level was 264.2 ± 4.384 pg/mg of protein in the Stattic-treated group and 273.7 ± 23.33 pg/mg of protein in the bvPLA2-treated group. These levels were significantly decreased to 199.4 ± 20.39 pg/mg of protein in the group treated with Stattic and bvPLA2 together. The β-secretase activity directly acting on Aβ production substantially increased with LPS treatment compared to that in the control. β-secretase activity was decreased by treating with Stattic or bvPLA2 and significantly decreased by co-treatment of Stattic and bvPLA2 (Fig. 7b). In addition, the increased expression levels of Aβ and BACE1 in BV-2 microglia due to LPS treatment were reduced by bvPLA2 treatment, but it was confirmed by Western blot that there was a greater decrease when treated together (Fig. 7c).

To investigate the combination effect of Stattic and bvPLA2 on neuroinflammation, levels of inflammatory proteins and of pro-inflammatory cytokines in BV-2 cells were examined. The intracellular levels of inflammatory proteins such as iNOS and COX-2 increased by LPS were reduced by bvPLA2 or Stattic and further decreased when both were used (Fig. 7d). The expression levels of TNF-α, IL-1β and IL-6, which were increased by LPS treatment, decreased when treated with bvPLA2 or Stattic. When used together, the levels of TNF-α, IL-1β, and IL-6 were significantly lower than those with a single treatment of Stattic and bvPLA2 (Fig. 7e).

## Discussion

In the present study, we found that bvPLA2 improves genetically impaired memory and cognitive function in a Tg2576 AD mouse model. Tg2576 mice demonstrated increases in genes and proteins associated with inflammation and amyloidogenesis, but these were reduced by the administration of bvPLA2. Activated microglia and astrocytes are known to greatly contribute to the production of Aβ and the aggravation of AD [7]. In the brains of patients with AD, there are activated astrocytes and microglia, and their proliferation is accompanied by increased production of Aβ [37]. In addition, microglia and astrocytes activated by various stressors cause the production of Aβ [7]. Activated astrocytes demonstrate increased Aβ production through the overexpression of APP, and increased activities of β-secretase and γ-secretase [38]. In the present study, we found that bvPLA2 reduced Aβ production and the expression of related genes, as well as the activation of microglia and astrocytes. Thus, the inhibitory effect of bvPLA2 on the activation of microglia and astrocytes could be related to the reduction of Aβ generation through the blockage of related gene expression. It is unclear how bvPLA2 inactivates microglia and astrocytes; however, several studies have been conducted to elucidate this. Baek et al. showed that the number of reactive microglia activated by 1-methyl-4-phenyl-1,2,3,6-tetrahydopyridine administration was reduced by bvPLA2 treatment, which contributed to an increase in Treg population [26]. In our previous study, we demonstrated that bee venom inhibits the activation of microglia or astrocytes by engaging the NF-κB signaling pathway. Therefore, the inhibitory effect of bvPLA2 on NF-κB signal may be related to the inhibitory effects of bvPLA2 on the activation of microglia and astrocytes.

Microglia and astrocytes are the representative cells that are imperative in neuronal inflammation in the central nervous system’s immune system. Aβ could activate microglia and astrocytes and increase the production and secretion of pro-inflammatory cytokines [38]. Microglia and astrocytes chronically activated by Aβ release inflammatory chemokines and cytokines such as IL-1, IL-6, and TNF-α [11, 12]. High expression of pro-inflammatory cytokines is also one of the main characteristics of AD [39]. Studies have shown that TNF-α, IL-1β, and IL-6 are associated with the pathogenesis of AD, and IL-1β, in particular, promotes Aβ production and enhances iNOS activity in astrocytes or neurons [37, 39–41]. We found that bvPLA2 reduces the levels of pro-inflammatory cytokines such as TNF-α, IL-1β, and IL-6 in vivo and in vitro. In contrast, bvPLA2 increases the levels of anti-inflammatory cytokines such as IL-4 and TGF-β in vivo and in vitro. Although IL-10 is known to be an anti-inflammatory cytokine, the expression levels of IL-10 were increased in Tg2576 mice and LPS treated BV-2 cells, and the levels of IL-10 were decreased by treating with bvPLA2. Expression of iNOS and COX-2 associated with neuroinflammation were also reduced. Guillot-Sestier demonstrated that in an environment of IL-10 deficiency, phagocytes of Aβ by microglia increase, thus inhibiting the progression of AD by blocking Aβ fibril formation [42]. Chakrabarty also reported that adeno-associated virus-mediated expression of IL-10 causes Aβ accumulation and memory impairment in APP mice [43]. Considering these reports, it can be interpreted that decreasing the expression level of TNF-α, IL-1β, IL-6, and IL-10 by bvPLA2 inhibits Aβ accumulation and thus helps to alleviate AD. We found that when bvPLA2 was administered to Tg2576 mice, levels of p-STAT3 and p-ERK decreased significantly in the brain. We also demonstrated the possibility of binding of STAT3 and bvPLA2 through the results of the pull-down assay and docking study. Moreover, the results of luciferase activity assay showed that bvPLA2 contributes to the inhibition of STAT3 transcriptional activity. We found that bvPLA2 could bind to the LD of STAT3 using the docking study. We have demonstrated that bvPLA2 binds to LD of STAT3 and exerts anti-inflammatory effects using mutant forms of STAT3. bvPLA2 had anti-inflammatory effects in microglial BV-2 cells transfected with WT-STAT3 and DBD-null STAT3, but could not affect cells with LD-null STAT3, suggesting that bvPLA2 exerts a beneficial effect by acting on LD-null STAT3. Mertens reported that mutation in LD of STAT3 inhibits the phosphorylation of STAT3 and thus inhibits its function as a transcriptional factor [44].

In our previous study, we demonstrated that bee venom inhibits the activation of Janus kinase 2/STAT3 pathway [28], and there have been reports that STAT3 activates the transcription of BACE1, APP, presenilin-1, and γ-secretase, which strongly contribute to Aβ production [22, 45]. Reports have demonstrated that the STAT3 signaling pathway is activated in activated microglia and reactive astrocytes, therefore describing a correlation between STAT3 signaling and neuroinflammation [46–48]. In addition, these substances could reduce the population of activated microglia and astrocytes by inhibiting the STAT3 signaling pathway and could exert anti-neuroinflammatory effects [48, 49]. These substances simultaneously inhibited Aβ accumulation, thereby alleviating memory impairment raising the possibility of treating AD [48, 50]. As an example, we have demonstrated that (E)-2-methoxy-4-(3-(4-methoxyphenyl) prop-1-en-1-yl) phenol inhibits the STAT3 signaling pathway, alleviates neuroinflammation, inhibits Aβ accumulation, and eventually restores memory impairment and cognitive abilities in an AD mouse model [50]. Eufumi et al. demonstrated that proinflammatory cytokines such as IL-6 induce phosphorylation of STAT3 and that phosphorylated STAT3 continues to activate microglia [18]. Therefore, we hypothesized that bvPLA2, one of the major components of bee venom, may play a role in the anti-neuroinflammation and anti-amyloidogenesis through inactivation of microglia and astrocytes by the inactivation of STAT3 and, thus, may help to alleviate AD.

In this study, we have demonstrated that bvPLA2 alleviates neuroinflammation and amyloidogenesis and therefore could be helpful in the treatment of AD. We have shown that this effect of bvPLA2 occurs by inhibiting the phosphorylation of STAT3, which is deeply related to neuroinflammation and Aβ production. Therefore, the present study suggests that bvPLA2 may be useful in the treatment or prevention of AD.

## Conclusion

This study demonstrated that bvPLA2 mitigates amyloidogenesis and neuroinflammation in Tg2576 mice and BV2 cells by inhibiting STAT3 signaling pathway. Therefore, bvPLA2 has potential as a therapeutic molecule in AD characterized by amyloidogenesis and neuroinflammation.

## Additional file

Additional file 1: **Figure S1.** Inhibitory effect of bvPLA2 on Aβ-induced neuroinflammation in BV-2 cells. (DOCX 372 kb)

## Data Availability

The data and materials of this study are available from the corresponding authors for reasonable requests.

## Abbreviations

AD: Alzheimer’s disease
APP: amyloid precursor protein
Aβ: β-amyloid
BACE1: β-secretase 1
bvPLA2: Bee venom phospholipase A2
COX-2: Cyclooxygenase 2
GFAP: Glial fibrillary acidic protein
IBA-1: Ionized calcium binding adaptor molecule 1
IL: Interleukin
iNOS: Inducible nitric oxide synthase
STAT3: Signal transducer and activator of transcription-3
TNF-α: Tumor necrosis factor α

## References

[1] Alzheimer’s A (2018). Alzheimers Dement.

[2] Alzheimer’s A (2016). 2016 Alzheimer’s disease facts and figures. Alzheimers Dement.

[3] Alzheimer’s A (2015). 2015 Alzheimer's disease facts and figures. Alzheimers Dement.

[4] Harach T, Jammes F, Muller C, Duthilleul N, Cheatham V, Zufferey V, Cheatham D, Lukasheva YA, Lasser T, Bolmont T (2017). Administrations of human adult ischemia-tolerant mesenchymal stem cells and factors reduce amyloid beta pathology in a mouse model of Alzheimer’s disease. Neurobiol Aging.

[5] Choi JY, Hwang CJ, Lee HP, Kim HS, Han SB, Hong JT (2017). Inhibitory effect of ethanol extract of Nannochloropsis oceanica on lipopolysaccharide-induced neuroinflammation, oxidative stress, amyloidogenesis and memory impairment. Oncotarget.

[6] Hardy JA, Higgins GA (1992). Alzheimer’s disease: the amyloid cascade hypothesis. Science.

[7] Yan R, Vassar R (2014). Targeting the beta secretase BACE1 for Alzheimer’s disease therapy. The Lancet Neurology.

[8] Gu SM, Park MH, Hwang CJ, Song HS, Lee US, Han SB, Oh KW, Ham YW, Song MJ, Son DJ (2015). Bee venom ameliorates lipopolysaccharide-induced memory loss by preventing NF-kappaB pathway. J Neuroinflammation.

[9] Heppner FL, Ransohoff RM, Becher B (2015). Immune attack: the role of inflammation in Alzheimer disease. Nat Rev Neurosci.

[10] Heneka MT, Carson MJ, El Khoury J, Landreth GE, Brosseron F, Feinstein DL, Jacobs AH, Wyss-Coray T, Vitorica J, Ransohoff RM (2015). Neuroinflammation in Alzheimer’s disease. The Lancet Neurology.

[11] Querfurth HW, LaFerla FM (2010). Alzheimer’s disease. N Engl J Med.

[12] Zheng C, Zhou XW, Wang JZ (2016). The dual roles of cytokines in Alzheimer’s disease: update on interleukins, TNF-alpha, TGF-beta and IFN-gamma. Translational neurodegeneration.

[13] Choi Ji, Jang Jun, Son Dong, Im Hyung-Sik, Kim Ji, Park Joung, Choi Won, Han Sang-Bae, Hong Jin (2017). Antarctic Krill Oil Diet Protects against Lipopolysaccharide-Induced Oxidative Stress, Neuroinflammation and Cognitive Impairment. International Journal of Molecular Sciences.

[14] Choi JY, Yeo IJ, Kim KC, Choi WR, Jung JK, Han SB, Hong JT (2018). K284-6111 prevents the amyloid beta-induced neuroinflammation and impairment of recognition memory through inhibition of NF-kappaB-mediated CHI3L1 expression. J Neuroinflammation.

[15] Han JH, Ju JH, Lee YS, Park JH, Yeo IJ, Park MH, Roh YS, Han SB, Hong JT (2018). Astaxanthin alleviated ethanol-induced liver injury by inhibition of oxidative stress and inflammatory responses via blocking of STAT3 activity. Sci Rep.

[16] Son DJ, Zheng J, Jung YY, Hwang CJ, Lee HP, Woo JR, Baek SY, Ham YW, Kang MW, Shong M (2017). MMPP attenuates non-small cell lung Cancer growth by inhibiting the STAT3 DNA-binding activity via direct binding to the STAT3 DNA-binding domain. Theranostics.

[17] Yu L, Chen C, Wang LF, Kuang X, Liu K, Zhang H, Du JR (2013). Neuroprotective effect of kaempferol glycosides against brain injury and neuroinflammation by inhibiting the activation of NF-kappaB and STAT3 in transient focal stroke. PLoS One.

[18] Eufemi M, Cocchiola R, Romaniello D, Correani V, Di Francesco L, Fabrizi C, Maras B, Schinina ME (2015). Acetylation and phosphorylation of STAT3 are involved in the responsiveness of microglia to beta amyloid. Neurochem Int.

[19] Wan J, Fu AK, Ip FC, Ng HK, Hugon J, Page G, Wang JH, Lai KO, Wu Z, Ip NY (2010). Tyk2/STAT3 signaling mediates beta-amyloid-induced neuronal cell death: implications in Alzheimer’s disease. J Neurosci.

[20] Kwon SH, Han JK, Choi M, Kwon YJ, Kim SJ, Yi EH, Shin JC, Cho IH, Kim BH, Jeong Kim S (2017). Dysfunction of microglial STAT3 alleviates depressive behavior via neuron-microglia interactions. Neuropsychopharmacology.

[21] Wen Y, Yu WH, Maloney B, Bailey J, Ma J, Marie I, Maurin T, Wang L, Figueroa H, Herman M (2008). Transcriptional regulation of beta-secretase by p25/cdk5 leads to enhanced amyloidogenic processing. Neuron.

[22] Liu L, Martin R, Kohler G, Chan C (2013). Palmitate induces transcriptional regulation of BACE1 and presenilin by STAT3 in neurons mediated by astrocytes. Exp Neurol.

[23] Mousavi SM, Imani S, Haghighi S, Mousavi SE, Karimi A (2012). Effect of Iranian honey bee (Apis mellifera) venom on blood glucose and insulin in diabetic rats. J Arthropod Borne Dis.

[24] Linderoth L, Peters GH, Jorgensen K, Madsen R, Andresen TL (2007). Synthesis of sn-1 functionalized phospholipids as substrates for secretory phospholipase A2. Chem Phys Lipids.

[25] Ye M, Chung HS, Lee C, Yoon MS, Yu AR, Kim JS, Hwang DS, Shim I, Bae H (2016). Neuroprotective effects of bee venom phospholipase A2 in the 3xTg AD mouse model of Alzheimer’s disease. J Neuroinflammation.

[26] Baek H, Jang HI, Jeon HN, Bae H (2018). Comparison of administration routes on the protective effects of bee venom phospholipase A2 in a mouse model of Parkinson's disease. Front Aging Neurosci.

[27] Jung Kyung-Hwa, Baek Hyunjung, Shin Dasom, Lee Gihyun, Park Sangwon, Lee Sujin, Choi Dabin, Kim Woojin, Bae Hyunsu (2016). Protective Effects of Intratracheally-Administered Bee Venom Phospholipase A2 on Ovalbumin-Induced Allergic Asthma in Mice. Toxins.

[28] Jo M, Park MH, Kollipara PS, An BJ, Song HS, Han SB, Kim JH, Song MJ, Hong JT (2012). Anti-cancer effect of bee venom toxin and melittin in ovarian cancer cells through induction of death receptors and inhibition of JAK2/STAT3 pathway. Toxicol Appl Pharmacol.

[29] Kang YM, Chung KS, Kook IH, Kook YB, Bae H, Lee M, An HJ (2018). Inhibitory effects of bee venom on mast cell-mediated allergic inflammatory responses. Int J Mol Med.

[30] Zhang S, Liu Y, Ye Y, Wang XR, Lin LT, Xiao LY, Zhou P, Shi GX, Liu CZ (2018). Bee venom therapy: potential mechanisms and therapeutic applications. Toxicon.

[31] Kim JI, Yang EJ, Lee MS, Kim YS, Huh Y, Cho IH, Kang S, Koh HK (2011). Bee venom reduces neuroinflammation in the MPTP-induced model of Parkinson's disease. Int J Neurosci.

[32] Morris R (1984). Developments of a water-maze procedure for studying spatial learning in the rat. J Neurosci Methods.

[33] Lee YS, Lee CH, Bae JT, Nam KT, Moon DB, Hwang OK, Choi JS, Kim TH, Jun HO, Jung YS (2018). Inhibition of skin carcinogenesis by suppression of NF-kappaB dependent ITGAV and TIMP-1 expression in IL-32gamma overexpressed condition. J Exp Clin Cancer Res.

[34] Lee YJ, Choi DY, Choi IS, Kim KH, Kim YH, Kim HM, Lee K, Cho WG, Jung JK, Han SB (2012). Inhibitory effect of 4-O-methylhonokiol on lipopolysaccharide-induced neuroinflammation, amyloidogenesis and memory impairment via inhibition of nuclear factor-kappaB in vitro and in vivo models. J Neuroinflammation.

[35] Kam K, Duffy AM, Moretto J, LaFrancois JJ, Scharfman HE (2016). Interictal spikes during sleep are an early defect in the Tg2576 mouse model of beta-amyloid neuropathology. Sci Rep.

[36] Westerman MA, Cooper-Blacketer D, Mariash A, Kotilinek L, Kawarabayashi T, Younkin LH, Carlson GA, Younkin SG, Ashe KH (2002). The relationship between Abeta and memory in the Tg2576 mouse model of Alzheimer’s disease. J Neurosci.

[37] Sawikr Y, Yarla NS, Peluso I, Kamal MA, Aliev G, Bishayee A (2017). Neuroinflammation in Alzheimer’s disease: the preventive and therapeutic potential of polyphenolic nutraceuticals. Adv Protein Chem Struct Biol.

[38] Frost Georgia R., Li Yue-Ming (2017). The role of astrocytes in amyloid production and Alzheimer's disease. Open Biology.

[39] Su F, Bai F, Zhang Z (2016). Inflammatory cytokines and Alzheimer’s disease: a review from the perspective of genetic polymorphisms. Neurosci Bull.

[40] Sheng JG, Ito K, Skinner RD, Mrak RE, Rovnaghi CR, Van Eldik LJ, Griffin WS (1996). In vivo and in vitro evidence supporting a role for the inflammatory cytokine interleukin-1 as a driving force in Alzheimer pathogenesis. Neurobiol Aging.

[41] Stamouli EC (2016). Politis AM: [pro-inflammatory cytokines in Alzheimer’s disease]. Psychiatrike = Psychiatriki.

[42] Guillot-Sestier MV, Doty KR, Gate D, Rodriguez J, Leung BP, Rezai-Zadeh K, Town T (2015). Il10 deficiency rebalances innate immunity to mitigate Alzheimer-like pathology. Neuron.

[43] Chakrabarty P, Li A, Ceballos-Diaz C, Eddy JA, Funk CC, Moore B, DiNunno N, Rosario AM, Cruz PE, Verbeeck C (2015). IL-10 alters immunoproteostasis in APP mice, increasing plaque burden and worsening cognitive behavior. Neuron.

[44] Mertens C, Haripal B, Klinge S, Darnell JE (2015). Mutations in the linker domain affect phospho-STAT3 function and suggest targets for interrupting STAT3 activity. Proc Natl Acad Sci U S A.

[45] Chiba T, Yamada M, Aiso S (2009). Targeting the JAK2/STAT3 axis in Alzheimer’s disease. Expert Opin Ther Targets.

[46] Huang C, Ma R, Sun S, Wei G, Fang Y, Liu R, Li G (2008). JAK2-STAT3 signaling pathway mediates thrombin-induced proinflammatory actions of microglia in vitro. J Neuroimmunol.

[47] Xia XG, Hofmann HD, Deller T, Kirsch M (2002). Induction of STAT3 signaling in activated astrocytes and sprouting septal neurons following entorhinal cortex lesion in adult rats. Mol Cell Neurosci.

[48] Zhang ZH, Yu LJ, Hui XC, Wu ZZ, Yin KL, Yang H, Xu Y (2014). Hydroxy-safflor yellow a attenuates Abeta(1)(−)(4)(2)-induced inflammation by modulating the JAK2/STAT3/NF-kappaB pathway. Brain Res.

[49] Paris D, Beaulieu-Abdelahad D, Abdullah L, Bachmeier C, Ait-Ghezala G, Reed J, Verma M, Crawford F, Mullan M (2013). Anti-inflammatory activity of anatabine via inhibition of STAT3 phosphorylation. Eur J Pharmacol.

[50] Choi JY, Hwang CJ, Lee DY, Gu SM, Lee HP, Choi DY, Oh KW, Han SB, Hong JT (2017). (E)-2-Methoxy-4-(3-(4-methoxyphenyl) prop-1-en-1-yl) phenol ameliorates LPS-mediated memory impairment by inhibition of STAT3 pathway. NeuroMolecular Med.

