# Supplementary material for: Bee venom phospholipase A2 ameliorates amyloidogenesis and neuroinflammation through inhibition of signal transducer and activator of transcription-3 pathway in Tg2576 mice
Source: Transl Neurodegener. 2019 Oct 2;8:26. doi: 10.1186/s40035-019-0167-7 (PMC6774221; doi:10.1186/s40035-019-0167-7)
Supplement: Supplementary file 1 — Figure S1. Inhibitory effect of bvPLA2 on Aβ-induced neuroinflammation in BV-2 cells. (DOCX 372 kb) [file 40035_2019_167_MOESM1_ESM.docx]

**Supplementary information for**

**Bee venom phospholipase A2 ameliorates amyloidogenesis and neuroinflammation through inhibition of signal transducer and activator of transcription-3 pathway in Tg2576 mice**

Hyeon Joo Ham^1^, In Jun Yeo^1^, Sang-Bae Han^1^, Jaesuk Yun^1^, Young Wan Ham^2^, Se Hyun Kim^3^, Pil-Hoon Park^4^, Dong-Young Choi^4^ and Jin Tae Hong^1^

^1^College of Pharmacy and Medical Research Center, Chungbuk National University, Osongsaengmyeong 1-ro, Osong-eup, Heungdeok-gu, Cheongju, Chungbuk, 28160, Republic of Korea

^2^Department of Chemistry, Utah Valley University, 800 W University Pkwy, Orem, UT 84058, USA^3^INIST ST CO., LTD^4^College of Pharmacy, Yeungnam University, 280 Daehak Road, Gyeonsan, Gyeongbuk 38541, Republic of Korea

E-mail addresses: HJH (prodijoo0918@nate.com), IJY (section18@naver.com), SBH (shan@chungbuk.ac.kr), JY (jyun@chungbuk.ac.kr), YWH (YHam@uvu.edu), SHK (sean0101@inistst.com), PHP (parkp@yu.ac.kr), DYC (dychoi@yu.ac.kr) and JTH (jinthong@chungbuk.ac.kr)

*Correspondence: Dr. Jin Tae Hong (jinthong@chungbuk.ac.kr), College of Pharmacy and Medical Research Center, Chungbuk National University, Osongsaengmyeong 1-ro, Osong-eup, Heungdeok-gu, Cheongju, Chungbuk, 28160, Republic of Korea, Tel: +82-043-261-2813, Fax: +82-043-268-2732.

**Supplementary Figure S1. Inhibitory effect of bvPLA2 on Aβ-induced neuroinflammation in BV-2 cells**

**
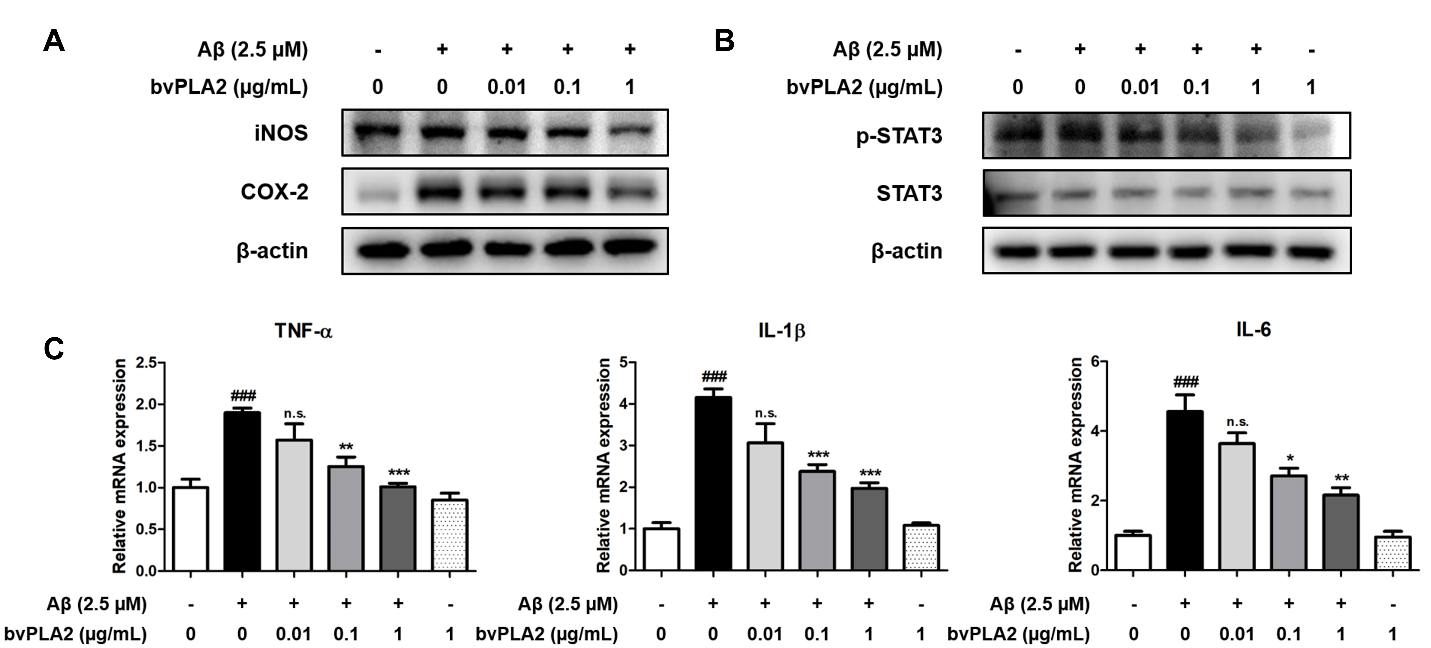
**

The expression of iNOS and COX-2 were detected by Western blotting using specific antibodies in BV-2 cells treated with bvPLA2 (A). The expression of p-STAT3 and STAT3 in BV-2 cells were detected by western blotting using specific antibodies (B). β-actin was used as a loading control. For the cropped images, samples were run in the same gels under same experimental conditions and processed in parallel. The mRNA expression level of pro-inflammatory cytokines (TNF-α, IL-1β, and IL-6) in BV-2 cells treated with bvPLA2 were assessed by qRT-PCR (C). Each data representative for three different experiments. Each value is mean ± S.E.M. from 3 samples. ###, Significantly different from control group (p < 0.001). *, Significantly different from Aβ group (p < 0.05). **, Significantly different from Aβ group (p < 0.01). ***, Significantly different from Aβ group (p < 0.001).
